# Targeting the Malaria Parasite cGMP-Dependent Protein Kinase to Develop New Drugs

**DOI:** 10.3389/fmicb.2020.602803

**Published:** 2020-12-17

**Authors:** David A. Baker, Alexios N. Matralis, Simon A. Osborne, Jonathan M. Large, Maria Penzo

**Affiliations:** ^1^Faculty of Infectious and Tropical Diseases, London School of Hygiene & Tropical Medicine, London, United Kingdom; ^2^”Alexander Fleming” Biomedical Sciences Research Center, Vari, Greece; ^3^LifeArc, Accelerator Building, Open Innovation Campus, Stevenage, United Kingdom

**Keywords:** malaria, cyclic GMP, signal transduction, *Plasmodium falciparum*, drug discovery

## Abstract

The single-celled apicomplexan parasite *Plasmodium falciparum* is responsible for the majority of deaths due to malaria each year. The selection of drug resistance has been a recurring theme over the decades with each new drug that is developed. It is therefore crucial that future generations of drugs are explored to tackle this major public health problem. Cyclic GMP (cGMP) signaling is one of the biochemical pathways that is being explored as a potential target for new antimalarial drugs. It has been shown that this pathway is essential for all of the key developmental stages of the complex malaria parasite life cycle. This gives hope that targeting cGMP signaling might give rise to drugs that treat disease, block its transmission and even prevent the establishment of infection. Here we review previous work that has been carried out to develop and optimize inhibitors of the cGMP-dependent protein kinase (PKG) which is a critical regulator of the malaria parasite life cycle.

## Introduction

Malaria kills more than 400,000 people each year, mainly young children in Africa (WHO, 2019), despite the availability of effective control measures and drug treatments. The selection and spread of drug resistant malaria parasites has made the development of new interventions an urgent priority so that increases in clinical cases and mortality can be avoided. High throughput screening of large chemical libraries has led to a number of promising new candidate antimalarial drugs that are progressing through clinical trials, but we cannot be sure how many of these will be rolled out as new treatments. It is therefore necessary that we continue to explore alternative drug targets that operate through different modes of action and progress the next generation of drugs to combat the serious problem of drug resistance.

One of the targets that has received significant attention in recent years is the cyclic GMP (cGMP)-dependent protein kinase (PKG) which is the central regulator of cGMP signaling in malaria parasites (Baker et al., 2017a; Cabrera et al., 2018). Cyclic GMP is an intracellular messenger molecule that is synthesized from GTP by guanylyl cyclase and broken down (hydrolyzed) by phosphodiesterases (PDEs). The cellular levels of cGMP are balanced by the opposing activities of these two enzyme classes which are in turn regulated by as yet unknown mechanisms. As cGMP reaches a threshold concentration in the cell, it binds to and activates PKG which can then phosphorylate its many intracellular protein substrates. The cGMP signaling pathway is present in most organisms and controls myriad processes across the animal kingdom ranging from transducing environmental signals (Bosgraaf and Van Haastert, 2002) and mediating locomotion in some protozoa (Linder et al., 1999) to controlling cardiovascular function (Bork and Nikolaev, 2018) and regulating phototransduction in humans (Stryer, 1986).

Protozoan parasites of the genus *Plasmodium* which cause malaria have a complex life cycle, with specialized forms adapted to both establishing infection and proliferation within the mammalian host and specific forms which mediate transmission to and from the *Anopheles* mosquito vector. We and others have demonstrated that cGMP signaling plays an essential role in all of the important stages of the *Plasmodium* life cycle (McRobert et al., 2008; Taylor et al., 2008, 2010; Moon et al., 2009; Falae et al., 2010; Collins et al., 2013; Brochet et al., 2014; Lakshmanan et al., 2015; Govindasamy et al., 2016; Koussis et al., 2020) indicating that targeting this pathway with a drug would interrupt the life cycle making it plausible to treat disease, block transmission and even to prevent infection. Cyclic GMP signaling through PKG is required to stimulate the mobilization of calcium from internal stores which in turn activates life cycle stage-restricted calcium-dependent protein kinases (CDPKs) to continue the signaling cascade and stimulate cell function through reversible protein phosphorylation (Dvorin et al., 2010; Brochet et al., 2014; Fang et al., 2018). PKG is also known to stimulate a protease cascade in blood stage parasites which it initiates by triggering the release of the subtilisin-like protease SUB1 from apical organelles called exonemes (Dvorin et al., 2010; Collins et al., 2013; Thomas et al., 2018). Numerous *P. falciparum* schizont proteins have been identified which require the activity of PKG to be phosphorylated (Alam et al., 2015), but it is not known how these mediate its role in merozoite egress.

## The Importance of a Small Gatekeeper Residue

A crucial structural feature of the parasite PKG underpins firstly the experimental approach that has been taken to study the function of PKG and secondly the utility of the enzyme as a drug target because this structural feature is not present in human PKG enzymes. This unique feature is simply the existence of a relatively small amino acid residue (threonine, Thr) in the so-called gatekeeper position of this protein kinase (McRobert et al., 2008). Human PKGs (and the majority of serine/threonine kinases) have a bulkier amino acid (e.g., Methionine, Met) at this position (Huang et al., 2010). The crucial consequence of having a small residue in this position is that it makes available a small hydrophobic pocket (the gatekeeper pocket) that adjoins the ATP-binding pocket (Donald et al., 2002; McRobert et al., 2008). Certain classes of small molecule ATP-competitive inhibitors can also occupy this small adjacent pocket with a protruding side chain which thereby confers exquisite inhibitor selectivity (Gum et al., 1998; Donald et al., 2002; Baker et al., 2017b). The presence of a bulky residue in the gatekeeper position prevents inhibitor access to the gatekeeper pocket and renders the kinase (and cell) insensitive to the inhibitor (Donald et al., 2002; McRobert et al., 2008). Exploitation of a kinase gatekeeper pocket to generate selective compounds has been used to tackle human disease by, for example, targeting p38α MAP kinase to inhibit cytokine-mediated inflammatory responses (Dominguez et al., 2005).

**FIGURE 1:**
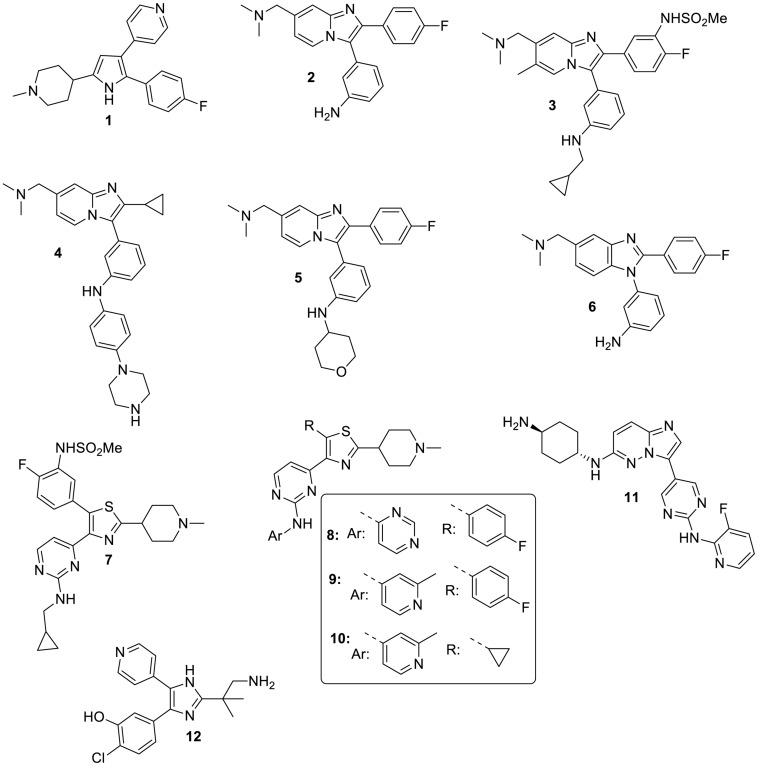
Representative structures of PKG inhibitors mentioned in the text.

**FIGURE 2:**
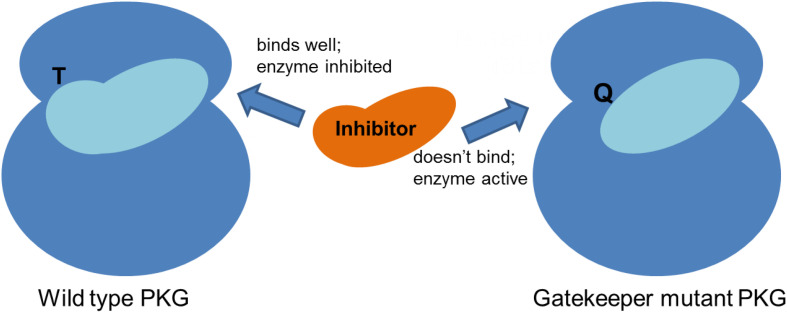
Schematic showing that inhibitors bind effectively to the malaria parasite PKG due to the presence of a small gatekeeper residue (threonine, T), which upon mutation to a bulkier residue (glutamine, Q), prevents inhibitor binding.

A research group at Merck working on the related parasite *Eimeria* (and the related genetically tractable *Toxoplasma*) first identified and utilized the small gatekeeper residue of apicomplexan PKGs to investigate the function of the enzyme in coccidian parasites (Donald et al., 2002) and importantly to demonstrate on-target activity of a potent anticoccidial agent they had identified by high throughput screening [a tri-substituted pyrrole, compound **1** (Gurnett et al., 2002)] and that of a more specific PKG inhibitor (an imidazopyridine, compound **2**, Figure 1) derived from a medicinal chemistry effort (Donald et al., 2006). Importantly, **1** was also shown to have inhibitory activity against malaria parasites (Diaz et al., 2006) and has (along with **2**) subsequently been used, in conjunction with transgenic lines expressing inhibitor resistant PKG alleles, to unequivocally determine the function of PKG throughout the malaria parasite life cycle (Baker et al., 2017a) (Figure 2). In the first such report, McRobert and colleagues generated a *Plasmodium falciparum* transgenic cell line in which the small gatekeeper residue (Thr618) was substituted with a bulkier glutamine (Gln) by allelic replacement at the endogenous *PKG* locus. Crucially, parasites expressing PKG with the bulky gatekeeper residue were several-fold less sensitive to compounds **1** and **2** than parental wild type parasites (McRobert et al., 2008). This approach of comparing the effects of a PKG inhibitor on wild type and gatekeeper mutant parasites has demonstrated an essential role for PKG in blood stage merozoite egress from red blood cells (Collins et al., 2013), release of male and female gametes from red blood cells (gametogenesis) (McRobert et al., 2008), maturation and motility of ookinetes (that develop following fertilization and zygote formation) (Moon et al., 2009; Brochet et al., 2014), motility of sporozoites and invasion of hepatocytes as well as release of merozoites from late liver stage parasites (Falae et al., 2010; Govindasamy et al., 2016). This combined chemical and genetic approach has demonstrated clearly that cGMP signaling mediated by PKG plays a vital role in a number of critical life cycle stage transitions and has simultaneously provided important data validating PKG as a tractable antimalarial drug target.

## PKG Inhibitors Often Have Structurally-Related Secondary Targets

As previously mentioned, the gatekeeper mutant approach has facilitated functional analysis of PKG in apicomplexan parasites and drug target validation for PKG. However, the approach also inherently allows determination of whether PKG is the primary target of a PKG inhibitor of interest and also whether there are significant secondary targets (Donald et al., 2002; Wiersma et al., 2004; McRobert et al., 2008). It is important to realise that IC_50_ values (derived from purified recombinant PKG) of compounds **1** and **2** for PKG are several logs different for wild type and gatekeeper mutant PKG, suggesting that these inhibitors do not easily access the gatekeeper pocket when a bulky gatekeeper residue is present. However, when EC_50_ values are compared (derived from e.g., blood stage growth inhibition assays) for different PKG inhibitors, it is clear that compared to IC_50_ levels, the difference is relatively low (several-fold rather than log-fold) (Donald et al., 2002; Baker et al., 2017b). This at first is perplexing, but actually reflects the fact that some PKG inhibitors have significant secondary targets that they bind to in the cell. This was first recognized in the earlier studies by the Merck team working on compounds **1** and **2** with *Eimeria* and *Toxoplasma* (Donald et al., 2006) who identified both CDPK1 and the alpha isoform of casein kinase 1 (CK1) as secondary targets of compound **2**. Importantly, it has proved possible by exploring Structure-Activity-Relationship studies (SAR) to generate PKG inhibitors with EC_50_ values that are also several logs different between wild type and gatekeeper parasites indicating that they are highly specific for PKG and that only very low levels of binding to secondary targets in the parasite and importantly the host occur (Baker et al., 2017b).

## PKG Antimalarial Drug Discovery Projects

Baker et al. (2017b) reported a PKG inhibitor development program using the Merck anticoccidial imidazopyridine compound **2** (ML1) as a start point. This resulted in the generation of more potent and selective analogs. The most promising compound **3** (ML10, Figure 1) has an IC_50_ value of 160 pM against recombinant wild type *P. falciparum* PKG and of 29.5 μM against gatekeeper mutant PKG in which threonine 618 was replaced by a glutamine. Activity against a panel of 80 human kinases (including 14 small gatekeeper kinases) was low when tested at 100 nM which is >600-fold the IC_50_ value against recombinant *P. falciparum* PKG. Of note, activity against human PKGI and PKGII isoforms was also very low as expected since they have bulky gatekeeper residues. **3** also had a clean *in vitro* cytotoxicity profile when tested against HepG2 cells and other mammalian cell lines. The EC_50_ value of **3** in 72-hour blood stage growth inhibition assays using wild type parasites (clone 3D7) was 2.1 nM and was >1,100-fold higher (EC_50_ = 2.4 μM) in gatekeeper parasites. This demonstrates that **3** is highly specific for PKG and that only very low levels of binding to secondary targets occurs which holds promise for developing a drug that selectively targets PKG. Furthermore, this compound has an EC_50_ of 41.3 nM in a standard membrane feeding assay (SMFA) which measures the ability of an inhibitor to block development of oocysts in mosquitos. The inhibitory activity reflects the known essential role of PKG in male and female gamete formation and in ookinete development and motility. Importantly this study also provided *in vivo* proof of concept for PKG as an antimalarial drug target by utilizing a *P. falciparum* humanized SCID mouse model which demonstrated that **3** reduced blood stage parasitaemia to undetectable levels. However, this was achieved by orally dosing with **3** twice daily for 4 days with 100 mg/kg which is a high dose compared with some other candidates undergoing pre-clinical and clinical testing.

To gain insight into the detailed molecular interaction of this chemical series with *Plasmodium* PKG and to inform future medicinal chemistry optimization efforts, **3** (ML10) and the Merck compound **2** (ML1) were each co-crystallized with *P. vivax* PKG. This protein proved more amenable to co-crystallization studies but is a good surrogate for the *P. falciparum* PKG because the orthologues share 92% sequence identity and alignment of the *apo* structures of the kinase domains show a deviation from each other by a root-mean-square distance of only 0.3 Å. (Baker et al., 2017b). The previously predicted interaction of the fluorophenyl moiety of the chemical series with the gatekeeper pocket (Donald et al., 2002) (on which the chemical genetic approach is based) was confirmed for both compounds and the high degree of selectivity of **3** (ML10) could be explained by the co-structures which included a more pronounced exploitation of the gatekeeper pocket and beyond by **3** than **2**, with the sulphonamide group of **3** extending into an additional cavity not reached by **2** (Baker et al., 2017b). The compound-free *P. falciparum* and *P. vivax* PKG full length structures obtained as part of this study were the first reported from any organism and contributed important insight into the mechanism by which the PKG is activated by cGMP (El Bakkouri et al., 2019).

One drawback of specific PKG inhibitors is that they are characterized by a moderate/slow killing rate in parasite reduction ratio (PRR) assays similar to that of pyrimethamine (Baker et al., 2017b; Matralis et al., 2019; Penzo et al., 2019). This is thought to be due to the fact that PKG is active within a very narrow temporal window of blood stage development (perhaps less than three hours) just prior to egress, which is thought to persist until completion of invasion (Taylor et al., 2010; Koussis et al., 2020). Specific PKG inhibitors have no activity against parasites outside of this window (Taylor et al., 2010).

Progress has also been reported to further explore and develop the imidazopyridine scaffold to improve key physicochemical parameters whilst retaining potency against blood stages. One aspect of this work focused on modification of the fluorophenyl moiety (that interacts with the gatekeeper pocket) of a set of bicyclic compounds and resulted in improved lipophilicity and *in vitro* ADME properties (Large et al., 2019a,b). For example, measured LogD could be improved from 2.4 (for compound **2**) to 1.5 in key analogs (compound **4**, Figure 1). In addition, stability was found to be moderate for compound **2** with 52% of this compound remaining after a 40-minute incubation with liver mouse microsomes. This liability was significantly improved by either appropriately substituting the amino group of **2** (compound **5**, Figure 1) or by applying scaffold hopping approaches (compound **6**, Figure 1). A parallel study also sought to optimize a set of monocyclic compounds (based on Merck trisubstituted pyrrole compound **1** that was developed to treat *Eimeria* infection in chickens), with the aims of improving structural alerts of the scaffold and increasing selectivity against human kinases. The design of the new molecules involved initially the replacement of the heteroaromatic pyrrole ring of **1** with its respective isostere thiazole followed by the replacement of the pyridine ring of **1** with that of 2-aminopyrimidine group existing in compound **2**. As a result, the trisubstituted thiazole series was generated. Excellent selectivity over human kinases was obtained in this series by modification of the 4-fluorophenyl group (Tsagris et al., 2018). The best selectivity was achieved by the 4-fluoro-3-sulfonamidophenyl group (compound **7**, Figure 1) which was also found to contribute significantly to the excellent PKG selectivity exhibited by **3**. Compound **7** did not show inhibition across a set of 80 human kinases when measured at a concentration 50 times higher than biochemical IC_50_ against recombinant *Pf*PKG. In addition, **7** showed an outstanding stability in both mouse and human microsomes (89 and 95% compound remaining, respectively, after 40 min of incubation), a remarkable aqueous solubility and LogD value (190 μM and 1.9, respectively) and good permeability (57 nm/s in the PAMPA assay).

To identify new chemical series that might overcome some of the limitations of existing PKG inhibitor scaffolds, a high throughput assay was developed to screen the GlaxoSmithKline Full Diversity collection of 1.7 million compounds in partnership with the Tres Cantos Open Lab Foundation. This identified nine clusters of interesting PKG inhibitor chemotypes including four clusters of trisubstituted heterocycles. The most interesting of these was a series of thiazoles that showed good potency against blood stage parasites and retained activity against gametogenesis as expected for compounds with low IC_50_ values against recombinant PKG enzyme activity. Hit expansion was performed using an available collection of more than 3,000 GSK thiazole derivatives. Measurement of EC_50_ values in 48- and 72-hour blood stage parasite growth inhibition assays indicated that some of the analogs might bind to additional targets apart from PKG. Specific PKG inhibitors exhibit a marked increase in EC_50_ in the 48-hour assay that reflects the narrow window of activity mentioned above. The existence of additional targets was confirmed using a chemoproteomic approach utilizing Kinobeads-based chemical proteomics. This identified additional targets including CDPK1, CDPK4, and NEK1 as additional targets for the thiazole derivatives studied (Penzo et al., 2019). Thiazoles have previously been developed by GSK as BRAF kinase inhibitors. For instance, Dabrafenib is used to treat a type of melanoma (Rheault et al., 2013) and it showed good selectivity against other human kinases with relatively mild toxicity (Falchook et al., 2014).

A medicinal chemistry project was subsequently carried out to further explore the potential of the thiazole series as antimalarials. The main goal of this research was to develop fast-kill agents given that the thiazole derivative **7** (Figure 1) developed in preceding studies still exhibits a moderately slow parasite killing rate. Toward this end, a chemical diversity SAR study was explored at all possible positions of the thiazole core ring, accompanied by isosterism approaches aiming at refining the structural determinants conferring fast-acting potency as well as favorable biopharmaceutical properties at the same time. Some of the compounds generated (compounds **8–10**, Figure 1) had a very fast speed of kill comparable to that of artesunate which is most likely due to inhibition of additional targets. Furthermore, **8–10** exhibited potent transmission blocking activity, attributed possibly to their potent PKG inhibitory activity, and desired ADMET properties (aqueous solubility, permeability, human serum albumin binding, metabolic stability). Compound **10** also showed an improved cytotoxicity (using HepG2 cells) and cardiotoxicity (*h*ERG inhibition) profile compared to compounds **7–9**. Again, Kinobeads profiling was used to identify these targets and the most likely candidate conferring the fast killing properties is a protein kinase called SRPK2 (CLK2) which is currently under investigation. This kinase and others identified in both chemoproteomic studies are amongst the relatively small number of *Plasmodium* kinases that have small gatekeeper residues which explains why they can bind to PKG inhibitors that exploit the gatekeeper pocket. These results raise the possibility of optimizing one of the thiazole leads **8–10** which combines the desirable properties of a PKG inhibitor with the fast killing properties conferred by inhibition of an additional target, likely CLK2 (Matralis et al., 2019).

In another study Green and colleagues reported a series of imidazopyridazine inhibitors that showed potent activity against the *P. falciparum* calcium-dependent protein kinase CDPK1 *in vitro* (Ansell et al., 2014). Class 1 of this series (with a pyrimidine linker between the core and the substituted amine) exhibited activity against late *P. falciparum* schizonts and one of the compounds (Compound **11**, Figure 1) had an EC_50_ value of 23 nM. However, the EC_50_ in the *P. falciparum* PKG T618Q gatekeeper mutant line was ∼20-fold higher than with wild type parasites revealing that the primary target of these compounds was actually PKG (Chapman et al., 2014; Green et al., 2015).

Recently, a team of scientists set out to identify the target of a tri-substituted imidazole (MMV030084, compound **12**, Figure 1) that was previously shown to have activity against *P. falciparum* blood stages in a high throughput screen of a GSK library of around two million compounds (Gamo et al., 2010). Further characterization of the activity profile showed that the compound had activity specifically against late stage *P. falciparum* blood stages with EC_50_ values of 109 and 120 nM against two different lab isolates, male gamete formation (EC_50_ = 141 nM) and invasion of hepatocytes by sporozoites (EC_50_ = 199 nM). A range of approaches including chemoproteomics and the use of transgenic *P. falciparum* parasites identified PKG as the primary target of this compound which is consistent with the known essential roles of PKG at all of these life cycle stages. A particularly interesting aspect of the study was that resistance-selection experiments with MMV030084 (**12**) did not generate highly resistant mutants (Vanaerschot et al., 2020). One might anticipate that highly resistant parasites could readily be generated by selection of a mutant under PKG inhibitor pressure where the gatekeeper threonine of PKG might be replaced by a bulkier residue. However, this did not prove to be the case in either this study or our own unpublished work in partnership with the Tres Cantos Open lab Foundation using the imidazopyridine ML10 (compound **3**, Figure 1). Reasons for this could include the fact that at least two base pairs would need to mutate to change the gatekeeper threonine to e.g., a glutamine and also that a threonine/glutamine substitution will likely have a fitness cost because although the PKG transgenic T618Q line is viable and can progress through the life cycle, we have shown that the kinetics of the PKG mutant enzyme are less favorable than wild type PKG with a ∼4-fold increase in K_m_ value for ATP but a similar V_max_ (Fang et al., 2018). Importantly, drug pressure using either ML10 (Cabrera et al., 2018) or MMV030084 (**12**) did not result in any mutation in the PKG sequence in either study. Vanaerschot and colleagues were able to select parasites with only low-level resistance to **12** which primarily relied on mutations in another protein kinase (tyrosine kinase-like protein 3) which is itself not a target of the compound (Vanaerschot et al., 2020). Together, the findings of this study strengthen the case for PKG as an antimalarial target worthy of additional study, not least because it appears to be a resistance-refractory target.

Apart from ATP-competitive inhibitors, cGMP analogs have also recently been explored as potential anti-malarials. It has been shown using biochemical and biophysical approaches that 8-NBD-cGMP, although having a similar affinity to cGMP, is a *P. falciparum* PKG antagonist with a 10-fold reduction of activation of PKG compared to cGMP (Byun et al., 2020). The effects on the parasite have so far not been reported.

## Conclusion

PKG has emerged as an exciting potential antimalarial drug target, but at a time when there are a number of other excellent targets that are being progressed and that are undergoing pre-clinical testing and clinical trials. Time will tell whether PKG inhibitors will be pursued in future pre-clinical testing and beyond and will likely depend on how many of the more advanced candidates are rolled out as components of new antimalarial combination treatments.

## Author Contributions

DB drafted the manuscript. SO, AM, JL, and MP co-wrote the manuscript. All authors contributed to the article and approved the submitted version.

## Conflict of Interest

The authors declare that the research was conducted in the absence of any commercial or financial relationships that could be construed as a potential conflict of interest.
